# Symptomatic treatment options for Huntington’s disease (guidelines of the German Neurological Society)

**DOI:** 10.1186/s42466-023-00285-1

**Published:** 2023-11-16

**Authors:** Carsten Saft, Jean-Marc Burgunder, Matthias Dose, Hans Heinrich Jung, Regina Katzenschlager, Josef Priller, Huu Phuc Nguyen, Kathrin Reetz, Ralf Reilmann, Klaus Seppi, Georg Bernhard Landwehrmeyer

**Affiliations:** 1 Department of Neurology, Huntington-Zentrum NRW, St. Josef-Hospital, Ruhr-Universität Bochum, Bochum, Germany; 2 Department of Neurology, Schweizerisches Huntington-Zentrum, Bern University, Bern, Switzerland; 3 Kbo-Isar-Amper-Klinikum, Taufkirchen/München-Ost, Germany; 4 Department of Neurology, Universitätsspital Zürich, Zürich, Switzerland; 5 Department of Neurology and Karl Landsteiner Institute for Neuroimmunological and Neurodegenerative Disorders, Klinik Donaustadt, Vienna, Austria; 6 Department of Psychiatry and Psychotherapy, Klinikum rechts der Isar, School of Medicine, Technical University of Munich, Munich, Germany; 7 Neuropsychiatry, Charité-Universitätsmedizin, Berlin, Germany; 8 Huntington-Zentrum NRW, Department of Human Genetics, Ruhr-Universität Bochum, Bochum, Germany; 9 Department of Neurology, Euregional Huntington Centre Aachen, RWTH Aachen University Hospital, Aachen, Germany; 10 George-Huntington-Institute, Muenster, Germany; 11 Department of Radiology, Universitaetsklinikum Muenster (UKM), Westfaelische Wilhelms-University, Muenster, Germany; 12 Department of Neurodegenerative Diseases and Hertie-Institute for Clinical Brain Research, University of Tuebingen, Tuebingen, Germany; 13 Department of Neurology, Medical University of Innsbruck, Innsbruck, Austria; 14 Department of Neurology, Huntington’s Disease Centre, Ulm University, Ulm, Germany

## Abstract

**Introduction:**

Ameliorating symptoms and signs of Huntington’s disease (HD) is essential to care but can be challenging and hard to achieve. The pharmacological treatment of motor signs (e.g. chorea) may favorably or unfavorably impact other facets of the disease phenotype (such as mood and cognition). Similarly, pharmacotherapy for behavioral issues may modify the motor phenotype. Sometimes synergistic effects can be achieved. In patients undergoing pragmatic polypharmacological therapy, emerging complaints may stem from the employed medications' side effects, a possibility that needs to be considered. It is recommended to clearly and precisely delineate the targeted signs and symptoms (e.g., chorea, myoclonus, bradykinesia, Parkinsonism, or dystonia). Evidence from randomized controlled trials (RCTs) is limited.

**Summary or definition of the topic:**

Therefore, the guidelines prepared for the German Neurological Society (DGN) for German-speaking countries intentionally extend beyond evidence from RCTs and aim to synthesize evidence from RCTs and recommendations of experienced clinicians.

**Recommendations:**

First-line treatment for chorea is critically discussed, and a preference in prescription practice for using tiapride instead of tetrabenazine is noted. In severe chorea, combining two antidopaminergic drugs with a postsynaptic (e.g., tiapride) and presynaptic mode of action (e.g., tetrabenazine) is discussed as a potentially helpful strategy. Sedative side effects of both classes of compounds can be used to improve sleep if the highest dosage of the day is given at night. Risperidone, in some cases, may ameliorate irritability but also chorea and sleep disorders. Olanzapine can be helpful in the treatment of weight loss and chorea, and quetiapine as a mood stabilizer with an antidepressant effect.

**Conclusions:**

Since most HD patients simultaneously suffer from distinct motor signs and distinct psychiatric/behavioral symptoms, treatment should be individually adapted.

## Introduction

This guideline is an abridged and translated short version of the guidelines of symptomatic treatment options for Huntington’s Disease of the German Neurological Society. A complete version of this guideline (in German) can be found on the website of the Deutsche Gesellschaft für Neurologie (www.dgn.org/leitlinien) and the AWMF (Arbeitsgemeinschaft wissenschaftlicher Medizinischer Gesellschaften).

AWMF registry No. Is 030/028; level of guideline: S2k; last update: November 24th, 2022; Valid until: November 23th, 2027; source: https://register.awmf.org/de/leitlinien/detail/030-028

This guideline has been approved by the German Neurological Society (DGN) and the German Association for Psychiatry, Psychotherapy, and Psychosomatics; German Society of Human Genetics; Swiss Neurological Society; Austrian Society of Neurology, and was reviewed by the German HD Association (Deutsche Huntington-Hilfe e.V.)

### What’s new?

Many individuals suffering from HD are oblivious to early as well as to progressing behavioral, cognitive, and motor symptoms and signs of the disease. Hence, it's advisable to conduct a comprehensive history that incorporates information gathered from a knowledgeable informant familiar with the patient/carrier of the Huntingtin gene expansion mutation (‘collateral history-taking’) whenever feasible. This lack of awareness seems to be a distinctive trait in HD and, in many cases, appears not to be linked to a deliberate avoidance of acknowledging symptoms or of suppressing signs (consensus strength 91%). Within this guideline, our objective was to provide insights to aid in enabling informed care decisions and to outline various potential treatment strategies addressing prevalent clinical matters covered in this guideline.

### Guidelines in detail

The recommendations of this guideline were established through a Delphi process (strength of consensus > 75–95% for all recommendations), achieved over two rounds of voting. All statements for which consensus strength is not specified separately were met with a consensus of > 95%.

#### Hyperkinesia/chorea

For the pharmacotherapy of choreatic hyperkinesia, D2/D3-dopamine receptor antagonists (e.g., tiapride) and inhibitors of vesicular monoamine Transporter 2 (VMAT2; e.g., tetrabenazine) are available. The quality of data from RCTs is currently best for VMAT2 inhibitors [18]. However, the tetrabenazine side effect of depression is clinically relevant since HD patients are prone to depression [37]. Comparative studies (comparing head-to-head compounds for their ability to suppress hyperkinesias short- and long-term) are unavailable. Therefore, no evidence-based recommendation can be made on best medical practice [9, 10, 31, 38, 43, 44].

For tiapride, which has been used since the 1970s for treating movement disorders (including HD), the lack of recent RCTs or clinical studies meeting today's quality standards is counterbalanced by many years of clinical real-life experience. Because of the favorable profile of side effects, in particular with regard to extrapyramidal (akathisia, Parkinsonism) and behavioral (depression) symptoms, the consensus in the Delphi group was that tiapride should be considered as first line treatment.

The use of tetrabenazine (in combination or as monotherapy) is recommended if treatment with tiapride is either not tolerated or not efficacious. Combining the two antidopaminergic drugs, tiapride (postsynaptic) and tetrabenazine (presynaptic), might help to reduce the dosage of the respective individual compounds and thereby reduce side effects. There are no studies on combination therapy available (see Table 1).

**Table 1:** List of compounds used to treat hyperkinesia in Huntington’s disease

Drug	Dosing	Important potential side effects
Tetrabenazine	Starting dose: 2 × 12,5 mg Target dose: up to 3 × 75 mg per day max. daily dose ~ 200 mg If sedative side effects occur, consider dividing the medication into four separate doses, with the primary dosage administered as the nighttime medication	Depression/suicidality, sedation, sleep disorders, and extrapyramidal side effects, Parkinsonism, akathisia Rare cases of a neuroleptic malignant syndrome Do not combine with MAO inhibitors!
Tiaprid	Starting dose: 2 × 50 mg Target dose: up to 4 × 300 mg pro Tag Max. Daily dose ~ 300 to 1000 mg. One study used up to 3000 mg daily If sedative side effects occur, consider dividing the medication into four separate doses, with the primary dosage administered at nighttime	Side effects similar to another classic dopamine D2 receptor antagonists, in particular sedation and Parkinsonism
Atypical neuroleptics, e.g. risperidone, olanzapine and aripiprazole	Similar to psychiatric indications	Only smaller studies and case reports Occasionally Parkinsonism monitoring for impulsivity and impulse control disorders for aripiprazole (Risperidone may also be helpful for the treatment of irritability; Olanzapine may also be beneficial for the treatment of weight loss)
Haloperidol	Similar to psychiatric indications 5 to 10 mg/day (as evening dose) sufficient in most cases	Side effects of a classic Dopamine receptor antagonists, esp. Parkinsonism not drug of the first choice possibly useful in cases with psychosis or aggressive behavior
Amantadine	100 to 400 mg daily dose (divided into 2 to 4 single doses)	Data partially contradictory Caution: Psychosis! (consensus strength 80%)
Valproate	effect dose-dependent	Rarely indicated in myoclonic Hyperkinesia (action myoclonus; [6, 10, 53])
Levetiracetam	Up to 2 × 1500 mg per day	Only small studies and case reports Parkinsonism, sedation, cognitive disorders, increased irritability!

Bonelli and Hofmann [9], Bonelli and Wenning [10], Brusa et al. [11], Ciammola et al. [14], Huntington Study [30], Killoran and Biglan [38], Mestre et al. [43, 44]

Alternatively, other antipsychotics can be used. Olanzapine (up to 30 mg/day) showed a favorable effect in two out of three small open studies [9, 10, 43, 44]. For quetiapine, zotepine, ziprasidone, and aripiprazole, only small studies and case reports describing a beneficial effect on motor function are available [9, 10, 43, 44]. A small study comparing the antichoreatic efficacy of aripiprazole and tetrabenazine describes a similar antichoreatic effect [11, 14]. For risperidone, a beneficial effect on motor and psychological functions after using a depot preparation was described [9, 10, 32, 43, 44]. This benefit may also be explained by an improvement of psychomotor restlessness, which may result in a beneficial effect on hyperkinesia. Clozapine did not reduce chorea [9, 10, 43, 44].

Many other studies investigating classical dopamine receptor antagonists (trifluperidol, thioproperazine, phenothiazine, trifluoperazine, perphenazine, chlorpromazine, melperone) reported no definite effects and these should not be used [10]. Haloperidol, however, has been shown to be effective in several Level II studies [9, 10, 43, 44].

Because of the potential to further slow down intended/voluntary movements and the fact that hypo-/brady-kinesia is a common sign in HD, all anti-choreic compounds should be used sparingly and should be prescribed primarily in the case of subjectively disabling hyperkinesias (if necessary, starting with very low dosages, e.g., only half a tablet; [26]). The impact of antichoreic prescriptions and, therefore, the success of an antichoreatic therapy may become apparent only with some delay, i.e., following a treatment period of four to six weeks for tiaprid and tetrabenazine. Therefore, an increase in dosage and dose reduction should always be done step by step, keeping in mind the delayed effects.

Given the frequently observed sedative side effects of compounds with antichoreatic efficacy, potentially aggravating a reversal of the day-night rhythm in extreme cases (consensus strength 91%), the principal dosage of antichoreatic drugs should be given as a night medication.

As a more hypokinetic-rigid phenotype often emerges in later stages of HD, a dose reduction of antidopaminergic compounds or switching to preparations with a lower risk for hypo-/brady-kinetic side effects might be helpful. A dose reduction of antidopaminergic drugs may also improve swallowing.

Data on the antichoreic efficacy of amantadine are partially contradictory, but amantadine appears to have an antichoreatic effect particularly in paraneoplastic chorea [10, 25

Levetiracetam has also been described as helpful [10; consensus strength 90%)].

Evidence for the effectiveness of memantine is lacking [47]. Ethyl-EPA showed no improvement in motor symptoms after six months of therapy [10]. A European phase III study confirms this negative result [19]. An improvement in the clinical signs and symptoms caused by the phosphodiesterase 10a inhibitor PF-02545920 could not be confirmed, the primary endpoint was not reached [17].

Cannabinoids such as Tetrahydrocannabinol and nabilone are considered potentially helpful in treating motor symptoms and signs [5, 13, 16]. Tetrahydrocannabinol was safe and well tolerated but without symptomatic effect in a randomized, double-blind cross-over placebo-controlled pilot study over twelve weeks of treatment ([46], consensus strength 90%).

Deep brain stimulation is experimental and should only be employed in the context of clinical studies. A small German pilot study on deep brain stimulation showed an improvement in chorea after GPi/GPe stimulation in some patients [63]. A complete analysis of an RTC on deep brain stimulation funded by DFG and the CHDI Foundation is underway. According to preliminary results, the primary endpoint of this clinical trial (UHDRS score at 12 weeks following surgery comparing stimulation on vs. stimulation off) was missed (presentation March 3, 2022, 17th Annual HD Therapeutics Conference, CHDI, Palm Springs; DRKS S00006785, Eudamed no. CIV-13-12-011770; consensus strength 90%). Chorea scores appeared to improve in both study arms in some participants, including the ones with stimulation off. A meta-analysis reported no significant changes in the UHDRS total score after restorative brain graft injections, whereas deep brain stimulation resulted in a substantial reduction of chorea. According to expert opinion in this metaanalysis, GPi-DBS should be considered in cases where the extent of chorea is the primary therapeutic challenge and should therefore be used selectively [39].

Deuterium-modified tetrabenazine (deutetrabenazine or SD-809) caused a reduction of chorea in a Phase III trial of the Huntington Study Group [30]. If and when this drug will be approved in Germany/Europe currently needs to be clarified. Likewise, valbenazine, which showed an improvement in chorea in the KINECT-HD study (NCT04102579), is not available in Europe/Germany at present (press release of December 8th, 2021; https://www.prnewswire.com/news-releases/neurocrine-biosciences-announces-positive-phase3-data-for-kinect-hd-study-evaluating-valbenazine-for-chorea-associated-with-huntington-disease-301439605.html).

#### Myoclonus

Valproate acid, in particular, but also clonazepam, levetiracetam, or piracetam, have been used to treat (action) myoclonus [6, 53].

#### Bruxism

Bruxism can occur as a side effect of neuroleptics or SSRIs but also as part of the natural history of HD [49]. A dose reduction of these drugs or a Botulinum toxin injection into the masseter muscle and a bite splint might help [6].

#### Dystonia

Treatment of dystonia in HD is challenging. Low-dose tetrabenazine has been suggested [35], as well as amantadine, baclofen, tizanidine, and clonazepam. There is no sufficient experience with Botulinum toxin injections or deep brain stimulation. Anticholinergics should be used with caution (e.g., on a probationary basis if side effects of antidopaminergic drugs are suspected), paying attention to possible cognitive or psychiatric side effects. A small case series describes a potential benefit of Cannabinoids on dystonia (e.g., Tetrahydrocannabinol or Dronabinol; [54]).

#### Hypo-/brady-kinesia, rigidity, tremor

Some case reports suggested an improvement under L-Dopa, amantadine, or pramipexole [10]. Dopamine agonists, in particular, may be helpful in bradykinetic patients and patients suffering from pediatric/juvenile onset HD where chorea may not be a feature. In some patients with difficulties in swallowing, improvements were reported following treatment with a rotigotine patch. As a possible side effect, drug-induced psychosis should be considered. In psychotic patients, Clozapine or Quetiapine are antipsychotics probably least likely to cause worsening of bradykinesia.

#### Complication: increased salivation

Increased salivation can be a side effect of antidopaminergic therapy and if dysphagia is present, this may increase the risk of developing aspiration pneumonia. A tricyclic antidepressant (e.g., amitriptyline or imipramine), pirenzepine, bornaprine hydrochloride (Sormodren), or a scopolamine patch may reduce salivation. Parasympatholytics, such as a few drops of atropine orally or botulinum toxin injections into the salivary glands, might be helpful. Anticholinergic drugs should be used cautiously because of potential cognitive or psychiatric side effects.

#### Depression

Depression is a common and clinically significant complaint in HD (consensus strength 91%). An increased rate of suicides is well documented, and the risk may be even higher in families with a history of (frequent) suicides. The time of clinical diagnosis (at the beginning of the disease) and the time when the condition has a functional impact threatening independent living are thought to be phases of increased suicidal risk. For that reason, suicidality should be assessed frequently [6]. Apathy and loss of motivation may be symptoms of depression, side effects of antidopaminergic drugs (pharmacogenic depression) or may occur independently of pharmacotherapy as part of the natural history of HD. Apathy typically increases over the course of the disease.

There is hardly any data from RCTs or controlled studies on treating mental health problems in HD. Recommendations are therefore based on expert opinion. Therapy, in general, is not different from the principles of psychiatric treatment. MAO inhibitors should be avoided (e.g. contraindication for tetrabenazine).

SSRIs and, in particular, venlafaxine [29] seem to be effective in patients suffering from severe depressive episodes. In patients with depression and sleep disorders, which may possibly be a part of the neurodegenerative process, the use of mirtazapine and mianserin [6], possibly also melatonin or melatonin agonists [1, 61] is recommended. Potent anticholinergic tricyclic antidepressants should be avoided, or only low dosages should be used because of potential side effects on hyperkinesias and cognition. However, tricyclic antidepressants might be helpful in patients suffering from sleep disturbances and simultaneously from hypersalivation.

In patients suffering from mild depression, antidepressant treatment with sulpiride (50–600 mg per day), a nearly selective D2 antagonist, which tends to improve hyperkinesia, might be helpful. Due to the inhibition of mainly presynaptic dopamine D2 receptors in the low dose range of up to 200 mg/day, effects on motivation are possible. To date, there have been few RCTs on antidepressant therapy for HD. A small randomized, double-blind, placebo-controlled study of fluoxetine in nondepressed patients with HD was negative for changes in total functional capacity (TFC) and standardized neurological, cognitive, and behavioral ratings [15]. There is limited experience with tianeptine, potentially helpful in anxious depression and somatizing complaints. In an animal model for HD, tianeptine improved synaptic plasticity in the hippocampus [2, 64].

In patients suffering from severe, therapy-refractory depressive episodes, electroconvulsive therapy (ECT) has been used [6]. However, ECT may cause massive (consensus strength 91%) short-term memory impairment and should, therefore, only be used exceptionally.

#### Apathy

Apathy is HD's most common psychiatric symptom [60]. The Track-HD study found a correlation between apathy and disease progression [57]. So far, there is no evidence-based treatment for apathy in HD. In individual patients, bupropion and modafinil were used. However, a phase II study (NCT01914965) could not demonstrate bupropion's efficacy as a treatment for apathy [22]. Lack of motivation can be a symptom of depression or a side effect of antidopaminergic compounds (pharmacogenic depression; see above). Apathy should be discussed with relatives as a clinical feature of HD as part of the psychoeducative information provided to assist in getting along with an HD patient in daily life. Individualized cognitive training and structured daily routines are recommended [3, 6].

A small open-label study with 16 participants reported improvements in depression and apathy, as well as improvement in some cognition tasks during treatment with cariprazine, an atypical neuroleptic and D3-selective D2/D3 receptor partial agonist ([45]; consensus strength 91%).

#### Obsessive–compulsive symptoms

In the context of HD, most patients don’t display characteristic compulsions according to ICD-10 definitions. In HD, repetitive behaviors often represent perseverations, "sticking" to specific mental processes and daily routines; in contrast, an urge to repeat actions or an increase of inner tension (required for a diagnosis of “compulsions”) if a compulsive desire is not pursued are rarely reported. So far, no controlled therapeutic studies have been published. Treatment attempts with SSRIs (especially in the case of anxiety), antipsychotics (e.g., olanzapine and risperidone), mood stabilizers, and clomipramine [3], as well as psychotherapeutic or behavioral therapy in individual cases, can be considered [6]. Amantadine was described as helpful in one patient with severe perseverations [38].

#### Fear, agitation

In patients with mild symptoms, herbal remedies, anxiolytics such as buspirone, hydroxyzine, non-tricyclic antidepressants (e.g., mirtazapine), and sedating antipsychotics with low anticholinergic side effect profile can be used.

For anxious depressive symptoms, using an SSRI or SNRI, such as venlafaxine, may be helpful (Caution is advised: in particular, initially, a worsening of symptoms is possible; [6]. With amitriptyline and diazepam in HD [10] and with opipramol, improvements of anxiety and somatoform symptoms have been reported [62]. As with every tricyclic substance, anticholinergic side effects must be considered. Based on a benefit-risk assessment, the use of benzodiazepines or benzodiazepine receptor agonists (zolpidem, zopiclone) is justified, especially in more advanced stages of the disease. The development of tolerance and rebound effects must be considered.

#### Irritability, aggressive behaviour, sexual disorders, agitated behavior

Irritability and aggression are common issues in the care for patients with HD. Improvements were reported after treatment with antipsychotics (particularly risperidone), valproate, benzodiazepines, beta-blockers (propranolol), SSRI, possibly in combination with mirtazapine or mianserin, as well as buspirone [6, 10, 20, 37, 51, 59]. The use of synergistic effects might be helpful. Risperidone, in some cases, not only reduces irritability but also chorea and improves sleep disruption. Olanzapine can also be beneficial for weight loss and chorea treatment, and quetiapine can also be used as a mood stabilizer with an additional antidepressant effect.

It should be noted that SSRIs, especially at the beginning of therapy, can worsen inner tension and irritability. In such a case, the additional administration of a benzodiazepine for 1–2 weeks might be helpful [3]. In addition, mood-stabilizing drugs and lamotrigine, lithium, or carbamazepine were described to be helpful [20, 38, 41, 56].

Zuclopenthixol (drops or depot), levomepromazine, and haloperidol [20, 27, 41] might be helpful in severe irritability and aggression. RCTs for the treatment of irritability in HD are not available. There are only single case reports for using cannabinoids, such as Dronabinol [54].

Behavior management techniques are helpful in individual cases, such as staying separate instead of arguing in case of a conflict, distraction, and setting a routine. In addition, low-potency neuroleptics might be beneficial. Whether administering the inhalation neuroleptic drug loxapine is helpful needs to be sufficiently explored.

Exhibitionism, which rarely occurs, was successfully treated in one case with leuprorelin. Hypersexuality has been reported to be treated with medroxyprogesterone, cyproterone acetate (note: increased risk of meningioma) in other dementias, and with atypical neuroleptics (as part of impulse control disorders). SSRI (high dose, if in the context of perseverative thinking and behavior) and clomipramine might be helpful [10, 33, 36, 58].

Often a depressive episode can cause reduced libido. On the other hand, sexual dysfunction is a well recognized potential side effect of SSRIs [6].

In agitated behavior, causes should be looked for, such as pain, constipation, voiding problems resulting in an overly filled urinary bladder, or environmental factors (stimulus shielding recommended; [6]).

#### Psychoses

Psychotic symptoms should be treated with antipsychotics. RCTs and formal studies on treatment for psychosis in HD are still missing. There is clinical experience with haloperidol, olanzapine, aripiprazole, risperidone, quetiapine, clozapine and amisulpride. Extrapyramidal motor side effects may occur, especially after treatment with amisulpride, haloperidol, and risperidone (high dose). Clozapine can be used in severe psychosis because this drug causes fewer extrapyramidal motor side effects (especially in bradykinetic, juvenile HD patients). Precautions to allow for early detection of life-threatening side effects (e.g., regular blood count checks; rules of "controlled use") need to be implemented [3].

#### Dementia

So far, the lack of RCTs implies that no evidence-based therapeutic recommendations can be given. There is no evidence supporting the use of memantine. Cholinesterase inhibitors were ineffective [9, 10, 42–44]. Latrepirdin (Dimebon) showed no improvement in cognition or function [24].

Speech therapy and occupational therapy with cognitive training might possibly be helpful [6].

#### Sleep disorders

About two-thirds of all HD patients suffer from sleep disorders. The etiology is diverse. Some patients may suffer from a sleep disorder in the context of a depressive episode. These patients should be treated with sedating antidepressants (without pronounced anticholinergic side effects) at night.

Sometimes sleep disorders occur because of sedative side effects of antihyperkinetic medication during daytime, with a day-night reversal. In this situation, it might be helpful to use sedative side effects of these drugs to induce sleep if the primary dosage is administered at nighttime. This dosing regime can also be beneficial for treating disturbing hyperkinesias during the night.

For disease-related alterations in circadian rhythm, melatonin, or sedating antidepressants such as mirtazapine and mianserin, trazodone, or antihistamines might be helpful. In milder cases, herbal remedies (e.g., valerian, lemon balm, passion flower, lavender) can be used. Hypnotics should be avoided or used only for a short time because of the possibility of habituation [3, 6].

#### Weight loss, difficulties in swallowing, vomiting, constipation

HD patients are frequently catabolic and therefore require high-caloric supplementation food, possibly up to six to eight meals per day and/or high-caloric food supplements. In the case of difficulties in swallowing, thickening liquids can be helpful. Under certain circumstances, an early percutaneous endoscopic gastrostomy (PEG) system might improve quality of life. Discussions early on with those affected and their relatives about the potential need for a PEG system are recommended. Recurrent vomiting and reflux may be seen in patients on PEG nutrition in the advanced stages of the disease due to a limited absorption capacity of the stomach. Continuous PEG nutrition using an infusomat, dilution of the high-caloric food with water, or appropriate fractioning of meals into several smaller portions may be helpful. In addition, a slight elevation of the upper body—about 30 degrees—can be beneficial in the case of reflux-related vomiting. Furthermore, myocloniform diaphragmatic hyperkinesia may be the cause of recurrent sudden vomiting. In these cases, an increase in antihyperkinetic medication or valproate acid in myocloniform diaphragmatic hyperkinesia might be helpful. On the other hand, reducing neuroleptics can be beneficial in recurrent vomiting [6]. Always consider the side effects of pharmacotherapy as a possible cause. Proton pump inhibitors should be used in gastric reflux [4].

Constipation can occur as part of the involvement of the autonomous nervous system, as a side effect of pharmacotherapy, and because of being bedridden. Using laxatives and procedures to improve constipation might be necessary in these cases. High-fiber food (e.g., wheat bran, psyllium husks, flaxseed), adequate drinking (1.5–2 L per day), and physical activity are recommended. Patients should be advised that suppressing the urge to defecate should be avoided. Macrogols (e.g., Movicol), sodium picosulfate, bisacodyl, or prucalopride might be used for acute functional and chronic constipation.

#### Pain

Sensitivity to pain and temperature seems to be reduced in most HD patients. In patients with sudden changes in behavior or a sudden increase in hyperkinesias and psychomotor restlessness, nociceptive pain should be ruled out as a possible cause (e.g., fractures after falls or infections of the urinary tract, the gallbladder, or the teeth, including gingivitis). Dental treatments for patients at advanced stages of the disease often need to be performed under general anesthesia [6].

#### Sweating

Some patients complain that excessive sweating is a nuisance for them and their caregivers. As a first step, pharmacotherapy should be checked for possible side effects. Changes in hormone status or metabolic diseases, such as an overactive thyroid, infections, or psychological alterations, such as anxiety disorders and withdrawal symptoms, should be excluded. The symptoms might also exist as part of autonomic dysregulation. Sage extract (capsules or tea) may be helpful. Anticholinergic drugs or subcutaneous botulinum toxin A injections are rarely indicated.

#### Incontinence

Occasionally, patients with HD develop "precipitate micturitions," which is a sudden urination without warning and an inability to stop voiding before the bladder is completely empty. Anticholinergics typically do not help, while carbamazepine (200 mg/day) may be effective [10]. The subjective urge to urinate (combined with very frequent/constant (unsuccessful) trips to the toilet) is a common complaint at advanced stages of HD. Urological interventions are often unsuccessful. Behavioral therapy approaches (starting with, e.g., hourly trips to the toilet, gradual "stretching" of the interval) have been found to be helpful in an inpatient setting.

#### Other non-drug forms of therapy

In addition to pharmacotherapy, symptomatic treatment should include approaches like psychotherapy, psychosocial support, physiotherapy, occupational therapy, and speech therapy [6, 37, 48].

#### Physical therapy

Evidence shows that physical therapy interventions improve fitness, motor function, and gait in patients with HD [48]. Based on physiotherapy recommendations published in 2020, therapeutic approaches in HD patients may include the following:

Aerobic training, alone or in combination with strength training to improve fitness and motor skills, and supervised gait training to improve the spatial and temporal characteristics of gait. In addition, there is weak evidence for physical training improving balance, however without reduction of the frequency of falls. Inspiratory and expiratory breath training improves respiratory function and capacity. Training transfers, getting up from the ground, and providing strategies for nursing staff to participate in physical activity in mid-stage HD can improve performance. There is an expert consensus on the use of positioning devices, seat adjustments, and training of caregivers in the late stages of HD. This is supported by other publications describing an improvement in gait stability through physiotherapy [8].

#### Speech therapy

In HD patients, the ability to communicate worsens as the disease progresses [23]. Dysarthria and the risk of dysphagia develop [28]. Dysphagia is one of the most critical complications and is the leading cause of death in HD [40]. It should also be emphasized that instrumental diagnostics, e.g., a fiberoptic endoscopic swallowing examination (FEES), may be needed to assess dysphagia [34, 55]. Therefore, involving speech therapists in the multidisciplinary care of HD patients is essential. Patients with corticobulbar symptoms might benefit from speech training [12]. It also seems beneficial if these therapists discuss cases with caregivers, including those from different health care proffessions, offering advice on facilitating conversations and social interactions to optimize the care of HD patients.

#### Occupational therapy

Overall, only a few studies deal with occupational therapy in HD. Cognitive training might be helpful in patients with cognitive disorders [6]. Symptom-based psychosocial interventions such as psychological procedures, occupational therapy, and physical activity are recommended to stabilize and maintain everyday functions.

#### Rehabilitation

Inpatient and outpatient rehabilitation are perceived as helpful [50]. An intensified aerobic endurance training of twelve patients showed no improvement but stabilization of symptoms if compared to the natural history of the disease in a 26-week follow-up [21]. More extensive, controlled studies are needed to confirm these data because of the small cohort and a missing control group (i.e., without training) in this study.

#### Psychosocial care

Psychological support should be offered to cope with the disease or the result of molecular genetic testing. Moreover, there should be support from a social worker to navigate the complex bureaucracy to apply for and obtain financial assistance and support for care needs. Early in the disease process, it is recommended to document living wills and care directives (e.g., under special consideration of questions such as "planting a PEG," life-prolonging medical measures including reanimation and mechanical ventilation). It is recommended to advise the patient when drawing up such documents and to describe in as much detail as possible a living will and information about procedures the patient agrees to and those they do not agree to. Relatives need support and should be informed about supportive services (e.g., outpatient care, possibilities of "preventive care"). If necessary, psychotherapeutic support should be offered. Families should be informed about regional and national HD advocacy groups and patient self-help meetings.

#### Other supportive measures

These include Huntington chairs (Halesworth chair, ROM CareLine wheelchair), crash helmets, nest building (contact with the "bed roll" in the back can relieve restlessness), basal stimulation (soothing full body wash, breath-stimulating rubs [7]), weighted blankets, early dental care [52]. Also, unique care beds are available (e.g., Mecoso GmbH or CareLine [50]). In case of a tendency to fall and an unsteady gait, a four-wheeled walker may be helpful. The use of a walker should be discussed with a physiotherapist or occupational therapist and ideally tested in practice sessions, as patients may stumble over the walker [6]. As communication help, "eline spreekt" was found to be helpful in severe dysarthric or even anarthric HD patients: http://www.elinespreekt.nl/de/

## Data Availability

Not applicable.
